# Determinants of self-care practices in patients with breast cancer-related upper limb lymphedema: a cross-sectional analysis

**DOI:** 10.3389/fpubh.2026.1903671

**Published:** 2026-09-01

**Authors:** Zhongru Cao, Hongbo Huo

**Affiliations:** Department of Medical Oncology, Harbin Medical University Cancer Hospital, Harbin, Heilongjiang, China

**Keywords:** breast cancer-related upper limb lymphedema, Influencing factor analysis, lymphedema health belief questionnaire, lymphedema limb function-disability- health scale, self-care behavior

## Abstract

**Objective:**

To assess the level of self-care practices among patients with BCRL and explore factors associated with these practices.

**Methods:**

A total of 137 patients with BCRL who received treatment at our hospital between April 2022 and 2023 were included in this study. All participants met the predefined inclusion and exclusion criteria. Data were collected using a general information questionnaire, the Self-Care Behavior Scale for BCRL Patients, the Lymphedema Limb Function-Disability-Health Scale, and the Lymphedema Health Belief Questionnaire.

**Results:**

The total self-care behavior score of patients with BCRL was 71.69 ± 7.42, with a score index of 74.68%. The total limb function-disability-health score was 105.61 ± 35.76, with a score index of 32.92%. The total health belief score was 99.68 ± 11.23, with a score index of 79.74%. Hierarchical regression analysis showed that age, body mass index (BMI), surgical approach, total limb function-disability-health score, and total health belief score were significantly associated with self-care behavior (*P* < 0.05).

**Conclusion:**

Self-care practices among patients with BCRL were moderate and require further improvement. Age, BMI, surgical approach, upper limb function-disability-health status, and health belief level were associated with self-care behavior. Given the cross-sectional design, these findings should be interpreted as associations rather than causal relationships. Healthcare professionals should assess patients' upper limb function and health beliefs and provide targeted health education, rehabilitation guidance, and self-management support to improve self-care practices and quality of life in this population.

## 1 Introduction

Breast cancer is one of the most common malignant tumors among women and remains a major threat to women's health (1). Current treatment strategies for breast cancer mainly include surgery, lymph node dissection, radiotherapy, chemotherapy, endocrine therapy, and targeted therapy. Although these treatments have improved tumor control and survival outcomes, BCRL remains an important complication that can negatively affect patients' physical function, psychological wellbeing, and quality of life (2). BCRL occurs when lymphatic drainage of the upper limb is impaired after breast cancer treatment. It can lead to upper limb swelling, pain, discomfort, restricted movement, skin changes, and recurrent infection. In addition to physical symptoms, upper limb lymphedema may also cause psychological distress and social difficulties, further reducing patients' quality of life (3).

Self-care plays an important role in the long-term management of BCRL. Common self-care practices include upper limb protection, skin care, moderate-intensity exercise, compression therapy, symptom monitoring, and disease-related knowledge acquisition. These practices may help improve lymphatic drainage, reduce swelling and discomfort, and support daily disease management (4). However, previous studies (5) have shown substantial variation in self-care behavior among patients with BCRL, and these behaviors are associated with multiple demographic, clinical, functional, and psychological factors. Therefore, this study aimed to assess the level of self-care behavior among patients with BCRL and explore factors associated with these practices. Data were collected using structured questionnaires and validated assessment tools. By analyzing patients' general characteristics, upper limb function-disability-health status, and health beliefs, this study sought to provide evidence for identifying patients who may need more targeted self-management support. The findings of this study may improve understanding of self-care practices among patients with BCRL and support the development of individualized health education and rehabilitation strategies. These results may also provide practical evidence for healthcare professionals to strengthen patient education, promote self-management, and improve overall wellbeing in this population.

## 2 Objectives and methods

### 2.1 Study subjects

Convenience sampling was used to recruit patients with BCRL who received treatment at our hospital between April 2022 and 2023. In this study, BCRL referred specifically to lymphedema involving the upper limb after breast cancer treatment. Only patients with unilateral BCRL were included. Patients with bilateral upper limb lymphedema were not included. Patients with isolated trunk or chest wall lymphedema without upper limb involvement were also not included.

**Figure 1:**
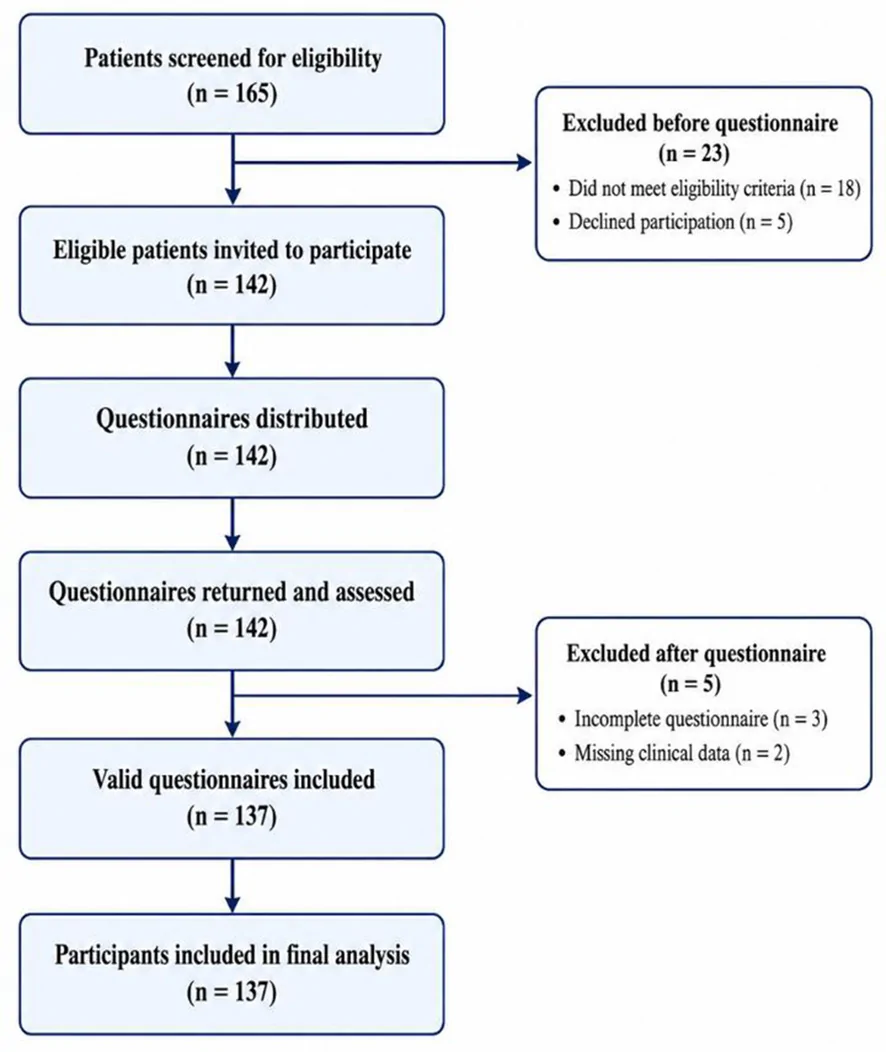
Flow diagram of participant screening, recruitment, questionnaire completion, and final analysis.

During the study period, 165 individuals were screened for eligibility. Among them, 23 individuals were excluded before questionnaire distribution, including 18 who did not meet the eligibility criteria and 5 who declined to participate. A total of 142 eligible patients were invited to participate, and 142 questionnaires were distributed. After questionnaire collection, 5 questionnaires were excluded because of incomplete questionnaire responses or missing clinical data. Finally, 137 valid questionnaires were included in the final analysis, with an effective response rate of 96.48%. The participant recruitment and enrollment process is shown in Figure 1.

The inclusion criteria were as follows: patients aged 18 years and above; female patients; patients clinically diagnosed with unilateral BCRL; patients with sufficient physical and cognitive ability to complete the questionnaire and with complete clinical data available for analysis; patients who were informed of the purpose of the study and provided written informed consent. The exclusion criteria were as follows: bilateral breast cancer, breast cancer recurrence, and distant metastasis; bilateral upper limb lymphedema; edema caused by cardiac, nutritional, and renal diseases; isolated trunk or chest wall lymphedema without upper limb involvement; severe heart failure, renal dysfunction, liver disease, and other serious systemic diseases; severe mental illness, cognitive impairment, incomplete clinical data, and refusal to participate in the study. This study was approved by the Ethics Committee of our hospital.

Ethics Statement: this study involving human participants was reviewed and approved by the Ethics Committee of Harbin Medical University Cancer Hospital. The study was conducted in accordance with the Declaration of Helsinki and local institutional requirements. Written informed consent was obtained from all participants before questionnaire administration. All data were anonymized before analysis, and no identifiable personal information was included in the manuscript.

### 2.2 Methods

#### 2.2.1 Research tools

The research tools used in this study included a general information questionnaire, the Self-Care Behavior Scale for BCRL Patients, the Lymphedema Limb Function-Disability-Health Scale, and the Lymphedema Health Belief Questionnaire. All three measurement scales were established instruments that had been developed or used in previous studies of BCRL or lymphedema management. Their reliability was considered acceptable in prior validation or application studies, and the Cronbach's α coefficients reported for the scales used in the present study were 0.847, 0.923, and 0.837, respectively. The detailed contents of these research tools are as follows:

**(1) General information:** The general information collected in this study included age, BMI, education level, marital status, place of residence, living alone status, employment status, monthly income, duration of upper limb lymphedema, and surgical approach.

**(2) Self-care behavior scale for BCRL patients** **(****6****):** This scale consists of five dimensions: activity and disease management, limb protection, skin care, disease knowledge, and the continuity of self-care, totaling 28 items. It uses a Likert four-point rating scale, ranging from “never” to “always” or from “completely disagree” to “completely agree,” scored from one to four points. Among them, 10 items are reverse-scored. The scale score ranges from 28 to 112 points, with higher scores indicating better self-management levels. The scale has been developed for assessing self-care behaviors in patients with BCRL, and the Cronbach's α coefficient in the present study was 0.847, indicating acceptable internal consistency. In this study, score indexes were used for analysis, defined as follows: good (score index ≥80%); moderate (score index in the range of 60–79%); poor (score index < 60%).

**(3) Lymphedema limb function-disability-health scale** **(****7****):** The lymphedema limb function-disability-health scale was mainly used to assess upper limb functional impairment in patients with BCRL and the impact of lymphedema on psychological function, household activity ability, mobility activity ability, life, and social function. It includes five dimensions: limb function, psychological function, household activity capability, mobility activity capability, and life and social function, totaling 29 items. Each item is rated using an 11-point Likert scale, with higher scores indicating greater severity of impact. The Chinese version of this scale has been psychometrically validated in patients with BCRL, and the Cronbach's α coefficient in the present study was 0.923, indicating good internal consistency. In this study, score indexes were used for analysis, defined as follows: moderately severe (score index ≤ 49%); severe (score index in the range of 50–89%); very severe (score index ≥90%).

**(4) Lymphedema health belief questionnaire** **(****8****):** This questionnaire is used to assess the level of self-management health beliefs in lymphedema patients. The questionnaire covers six dimensions: susceptibility, severity, benefits, barriers, self-efficacy, and cues to action, with a total of 26 items. It uses a Likert five-point rating scale, ranging from “strongly disagree” to “strongly agree,” scored from one to five points. The total questionnaire score and scores for each dimension are accumulated from the scores of all items, with a score range of 26- 130 points. Higher scores indicate stronger health management beliefs. This questionnaire has been used in lymphedema-related health belief and self-management research, and the Cronbach's α coefficient in the present study was 0.837, indicating acceptable internal consistency. In this study, score indexes were used for analysis, defined as follows: good (score index ≥80%); moderate (score index in the range of 60–79%); poor (score index < 60%).

#### 2.2.2 Data collection method

After eligibility was confirmed, trained investigators explained the purpose, procedures, and significance of the study to the patients. Written informed consent was obtained before questionnaire administration. Questionnaires were distributed and collected by two healthcare professionals who had received standardized training. For patients with reading difficulties, trained investigators conducted face-to-face interviews and assisted them in completing the questionnaires without guiding or influencing their responses. Completed questionnaires were checked on site for completeness. The detailed participant screening, questionnaire distribution, and final inclusion process is presented in Figure 1.

### 2.3 Statistical analysis

SPSS 23.0 was used for data analysis. Quantitative data were described as mean ± standard deviation and median (P25, P75), according to data distribution. Comparisons were performed using *t*-tests and analysis of variance. Categorical data were presented as frequencies and percentages, and comparisons were conducted using chi-square tests and Fisher's exact tests. Hierarchical multiple linear regression analysis was performed to explore variables associated with self-care behavior. The total self-care behavior score was used as the dependent variable. Independent variables were selected based on the study objectives, previous literature, clinical relevance, and preliminary univariate analyses. Variables were entered into the model in two blocks. Model 1 included age and BMI, which showed significant differences in the preliminary univariate analyses. Model 2 further added surgical approach, total limb function-disability-health score, and total health belief score to examine whether clinical characteristics, functional status, and health beliefs were associated with self-care behavior after controlling for age and BMI. The enter method was used within each block. Categorical variables were coded as dummy variables, as shown in Table 1. Model fit was assessed using *R*^2^, adjusted *R*^2^, changes in *R*^2^ (ΔR^2^), *F* values, and *P* values. A value of *P* < 0.05 was considered statistically significant.

**Table 1:** General information (*n* = 137).

Parameter	Number (*n*)	Composition (%)
Age (years)		
≤ 40	38	27.74%
41–59	65	47.44%
≥60	34	24.82%
BMI (kg/m^2^)		
< 19	4	2.92%
19.1–23.9	54	39.42%
24.0–27.9	57	41.60%
≥28	22	16.06%
Education level		
High school and below	54	39.42%
College and above	83	60.58%
Marital status		
Married	117	85.40%
Unmarried	20	14.60%
Residence		
Urban	127	92.70%
Rural	10	7.30%
Living alone		
Yes	5	3.65%
No	132	96.35%
Employment status		
Employed	58	42.34%
Unemployed	79	57.66%
Monthly income (CNY)		
≤ 5,000	66	48.18%
>5,000	71	51.82%
Lymphedema duration (years)		
< 1	73	53.28%
1–3	39	28.47%
>3	25	18.25%
Surgery type		
Modified radical mastectomy	78	56.93%
Lumpectomy + sentinel lymph node biopsy	3	2.19%
Lumpectomy + axillary lymph node dissection	56	40.88%

BMI, body mass index; CNY, Chinese Yuan. Lymphedema duration refers to the time from the diagnosis or onset of BCRL to questionnaire completion.

## 3 Results

### 3.1 General information

A total of 137 patients with BCRL were included in this study. The mean age of the participants was 49.70 ± 11.36 years, and the mean BMI was 24.83 ± 3.24 kg/m^2^. The detailed demographic and clinical characteristics of the study population are presented in Table 2.

**Table 2:** Scores of questionnaires and corresponding subscales in patients with BCRL.

Questionnaire/scale	Dimension	Number of items (*n*)	Mean score (points)	Total score (points)	Score index (%)
Self-care behavior scale for BCRL patients	Activity and disease management	10	1.75 ± 0.53	17.46 ± 5.08	_−_
Self-care behavior scale for BCRL patients	Limb protection	7	3.54 ± 0.47	24.56 ± 3.17	_−_
Self-care behavior scale for BCRL patients	Skin care	4	2.43 ± 0.75	9.68 ± 3.11	_−_
Self-care behavior scale for BCRL patients	Disease knowledge	4	2.59 ± 0.56	10.23 ± 2.35	_−_
Self-care behavior scale for BCRL patients	Self-care continuity	3	3.32 ± 0.64	9.86 ± 2.04	_−_
Self-care behavior scale for BCRL patients	Total self-care behavior score	28	2.58 ± 0.29	71.69 ± 7.42	74.68%
Lymphedema limb function-disability-health scale	Limb function	7	3.03 ± 1.89	21.32 ± 8.09	_−_
Lymphedema limb function-disability-health scale	Psychological function	4	3.35 ± 2.41	13.60 ± 8.73	_−_
Lymphedema limb function-disability-health scale	Household activity ability	4	3.67 ± 2.62	13.92 ± 9.34	_−_
Lymphedema limb function-disability-health scale	Mobility activity ability	7	4.43 ± 1.79	29.83 ± 10.51	_−_
Lymphedema limb function-disability-health scale	Life and social function	7	3.36 ± 2.01	22.42 ± 13.95	_−_
Lymphedema limb function-disability-health scale	Total limb function-disability-health score	29	3.64 ± 1.45	105.61 ± 35.76	32.92%
Lymphedema health belief questionnaire	Susceptibility	3	3.86 ± 0.82	11.68 ± 2.47	_−_
Lymphedema health belief questionnaire	Severity	5	4.27 ± 0.65	21.19 ± 3.16	_−_
Lymphedema health belief questionnaire	Benefits	4	4.34 ± 0.61	17.43 ± 2.38	_−_
Lymphedema health belief questionnaire	Barriers	7	3.07 ± 0.80	21.69 ± 5.53	_−_
Lymphedema health belief questionnaire	Self-efficacy	3	4.04 ± 0.82	12.07 ± 2.45	_−_
Lymphedema health belief questionnaire	Cues to action	4	3.95 ± 0.63	15.76 ± 2.42	_−_
Lymphedema health belief questionnaire	Total health belief score	26	3.87 ± 0.45	99.68 ± 11.23	79.74%

For the Self-Care Behavior Scale for BCRL Patients, higher scores indicate better self-care behavior. For the Lymphedema Limb Function-Disability-Health Scale, higher scores indicate more severe upper limb functional impairment and greater impact on daily life and social function. For the Lymphedema Health Belief Questionnaire, higher scores indicate stronger health beliefs related to lymphedema self-management. Score index was calculated as the actual score divided by the maximum possible score × 100%.

### 3.2 Scores on the self-care behavior scale, lymphedema limb function-disability-health scale, and lymphedema health belief questionnaire in patients with BCRL

The scores on the BCRL Patient Self-Care Behavior Scale, Lymphedema Limb Function-Disability-Health Scale, and Lymphedema Health Belief Questionnaire for breast cancer-related lymphedema patients are presented in Table 3 below.

**Table 3:** Comparison of self-care behavior scores among breast cancer-related lymphedema patients with different characteristics (*n* = 137).

Parameter	Self-care score (points)	t/Z	*P*
Age (years)		3.135	0.043
≤ 40	73.67 ± 7.15		
41–59	71.76 ± 7.24		
≥60	69.53 ± 6.91		
BMI (kg/m^2^)		3.536	0.019
< 19	68.54 ± 5.23		
19.1–23.9	73.05 ± 6.88		
24.0–27.9	72.13 ± 7.89		
≥28	67.64 ± 6.25		
Education level		0.022	0.982
High school and below	71.68 ± 7.82		
College and above	71.65 ± 7.59		
Marital status		0.362	0.717
Married	71.64 ± 7.11		
Unmarried	71.03 ± 5.86		
Residence		1.053	0.294
Urban	71.93 ± 7.35		
Rural	69.38 ± 7.67		
Living alone		0.805	0.422
Yes	75.32 ± 5.94		
No	72.59 ± 7.48		
Employment status		1.122	0.263
Employed	72.52 ± 6.33		
Unemployed	71.09 ± 8.04		
Monthly income (CNY)		0.500	0.617
≤ 5,000	72.37 ± 7.51		
>5,000	71.74 ± 7.22		
Lymphedema duration (years)		3.001	0.054
< 1	71.96 ± 6.88		
1–3	69.63 ± 7.52		
>3	73.81 ± 8.04		
Surgery type		2.486	0.083
Modified radical mastectomy	70.75 ± 7.08		
Lumpectomy + sentinel lymph node biopsy	67.63 ± 3.24		
Lumpectomy + axillary lymph node dissection	73.19 ± 7.82		

BMI, body mass index; CNY, Chinese Yuan. Self-care score refers to the total score of the Self-Care Behavior Scale for BCRL Patients. Higher scores indicate better self-care behavior. t/Z values were derived from t-tests or analysis of variance, as appropriate.

### 3.3 Comparison of self-care behavior scores among patients with BCRL with different characteristics

Statistically significant differences were observed in the self-care behavior scores among BCRL patients of different ages and BMI (*P* < 0.05). However, there were no statistically significant differences in self-care behavior scores based on other indicators (*P* > 0.05). The details are presented in Table 4 below.

**Table 4:** Assignment of values for independent variables.

Characteristic	Value assignment
Age (years)	≤ 40 = 1; 41–59 = 2; ≥60 = 3
BMI (kg/m^2^)	< 19 = 1; 19.1–23.9 = 2; 24.0–27.9 = 3; ≥28 = 4
Education level	High school or below = 1; College or above = 2
Marital status	Married = 1; Unmarried = 2
Residence	Urban = 1; Rural = 2
Living alone	Yes = 1; No = 2
Employment status	Employed = 1; Unemployed = 2
Monthly income (CNY)	≤ 5,000 = 1; >5,000 = 2
Lymphedema duration (years)	< 1 = 1; 1–3 = 2; >3 = 3
Surgery type	Dummy variables were used. Modified radical mastectomy was used as the reference category. Breast-conserving surgery + sentinel lymph node biopsy: yes = 1, no = 0. Breast-conserving surgery + axillary lymph node dissection: yes = 1, no = 0.
Limb function-disability-health total score	Entered as original continuous value
Health belief total score	Entered as original continuous value

BMI, body mass index. Dummy variables were used for categorical variables with more than two categories. Modified radical mastectomy was used as the reference category for surgical approach. Continuous scale scores were entered as original values.

### 3.4 Analysis of factors associated with self-care behavior in patients with BCRL

This study used hierarchical multiple linear regression analysis with the total self-care behavior score as the dependent variable. Variable selection was based on the study objectives, prior evidence, clinical relevance, and the results of preliminary univariate analyses. In Model 1, age and BMI were entered because they showed significant differences in univariate analyses. In Model 2, surgical approach was further entered because of its clinical relevance to lymphatic injury, rehabilitation needs, and self-care behavior in patients with BCRL. The total limb function-disability-health score and the total health belief score were also added in Model 2 to assess whether functional status and health beliefs were associated with self-care behavior after controlling for age, BMI, and surgical approach. Other collected demographic and clinical variables, including education level, marital status, residence, living alone status, employment status, monthly income, and lymphedema duration, were examined in preliminary analyses. These variables were not entered into the final hierarchical regression model because they did not show statistically significant associations with self-care behavior in the preliminary analyses and to maintain model parsimony. However, we acknowledge that education level, socioeconomic status, and lymphedema duration may still be clinically relevant and may have potential confounding effects. In addition, access to lymphedema-related care was not directly measured in this study and therefore could not be included in the regression model.

The results showed that Model 1 explained 13.2% of the variance in self-care behavior (*R*^2^ = 0.132, adjusted *R*^2^ = 0.119, *F* = 10.189, *P* < 0.001). After surgical approach, limb function-disability-health total score, and health belief total score were added, the explanatory power of Model 2 increased to 42.1% (*R*^2^ = 0.421, adjusted *R*^2^ = 0.399, *F* = 19.050, *P* < 0.001), with an additional explained variance of 28.9% (ΔR^2^ = 0.289, ΔF = 21.796, *P* < 0.001). In the final model, age, BMI, surgical approach, limb function-disability-health total score, and health belief total score were significantly associated with self-care behavior in patients with BCRL. The assignment of values for variables entered into the hierarchical regression model is presented in Table 1, and the regression results and model fit indicators are shown in Table 5.

**Table 5:** Hierarchical regression analysis results and model fit indicators.

Variables	*B*	β	*t*	*P*
Model 1				
Constant	84.735	_−_	14.413	< 0.001
Age	−2.234	−0.227	−2.105	0.038
BMI	−2.223	−0.231	−2.732	0.009
Model fit	*R*^2^ = 0.132	Adjusted *R*^2^ = 0.119	*F* = 10.189	< 0.001
Model 2				
Constant	65.052	_−_	9.574	< 0.001
Age	−2.141	−0.213	-2.169	0.036
BMI	−2.429	−0.256	-3.235	0.004
Surgery Type	2.617	0.172	2.079	0.043
Limb function-disability-health total score	0.221	0.325	3.962	< 0.001
Health belief total score	0.236	0.381	4.847	< 0.001
Model fit	*R*^2^ = 0.421	Adjusted *R*^2^ = 0.399	*F* = 19.050	< 0.001
Model change	ΔR^2^ = 0.289	ΔF = 21.796		< 0.001

*B*, unstandardized regression coefficient; β, standardized regression coefficient; *R*^2^, coefficient of determination; adjusted *R*^2^, adjusted coefficient of determination; ΔR^2^, change in *R*^2^ after adding variables to the model; F, overall model F statistic; ΔF, F statistic for model change. BMI, body mass index. *P* < 0.05 was considered statistically significant.

## 4 Discussion

### 4.1 Self-care behavior, upper limb function-disability-health status, and health belief levels need improvement in patients with BCRL

The results of this study showed that the total self-care behavior score of patients with BCRL was 71.69 ± 7.42, with a score index of 74.68%. The total score of the limb function-disability-health scale was 105.61 ± 35.76, with a score index of 32.92%, and the total score of the health belief scale was 99.68 ± 11.23, with a score index of 79.74%. These results suggest that self-care behavior, upper limb function-disability-health status, and health belief level in patients with BCRL still require improvement.

Previous research has shown that upper limb dysfunction in patients with BCRL may be associated with impaired axillary lymphatic drainage, lymph fluid accumulation, upper limb swelling, skin fibrosis caused by subcutaneous connective tissue proliferation, and increased susceptibility to upper limb infection (9). Once upper limb lymphedema becomes chronic and persistent, patients often require long-term self-management, which may substantially affect their daily life and work (10). Another study reported that functional impairment and activity limitation may further worsen upper limb dysfunction in patients with BCRL (11). The National Comprehensive Cancer Network guidelines also emphasize the importance of patient education and supportive care in the management of breast cancer-related complications (12). In addition, because most patients with BCRL spend much of their time at home, self-care behavior plays an important role in symptom relief and long-term disease management (13). Therefore, healthcare professionals should recognize the long-term impact of BCRL, strengthen health education, and provide targeted self-management support to help patients relieve edema symptoms, improve health beliefs, enhance home-based self-care ability, and improve quality of life.

### 4.2 Analysis of factors associated with self-care behavior in patients with BCRL

#### 4.2.1 Age and BMI

The results of this study showed that age and BMI were significantly associated with self-care behavior among patients with BCRL. Specifically, older age and higher BMI were associated with lower self-care behavior scores. This finding is consistent with previous studies (14, 15).

Several explanations may account for these findings. Older patients may experience declines in physical function, learning ability, and ability to perform daily self-care tasks. They may also have more difficulty accessing and using online health information, which may limit their understanding of lymphedema management and reduce adherence to self-care practices. In addition, patients with higher BMI may have reduced mobility, lower physical endurance, and greater difficulty performing moderate-intensity exercise, compression therapy, limb protection, and other self-care activities. Therefore, healthcare professionals should provide individualized rehabilitation plans for older patients and patients with higher BMI. Health education should be delivered in clear and accessible formats, with repeated guidance and follow-up support to improve patients' understanding, confidence, and adherence to self-care practices.

#### 4.2.2 Surgical approach

The results of this study showed that patients who underwent breast-conserving surgery combined with axillary lymph node dissection had higher self-care behavior scores than those who underwent modified radical mastectomy (*B* = 2.617, *P* = 0.043). Because this was a cross-sectional study, this finding should be interpreted as an association rather than evidence of a causal effect. One possible explanation is that breast-conserving surgery may involve less tissue trauma and lower psychological burden than modified radical mastectomy, which may help patients maintain better physical function and engage more actively in self-care practices (16). Therefore, healthcare professionals should assess patients' physical and psychological status when providing lymphedema management. For patients undergoing modified radical mastectomy, greater attention should be paid to psychological support, rehabilitation education, and continuous self-management guidance. Recent nursing studies also support the importance of perioperative and psychosocial care in patients with breast cancer. Zuo et al. (17) reported that perioperative comfort care improved postoperative recovery in patients with breast cancer, suggesting that comfort-oriented nursing may help reduce postoperative discomfort and promote rehabilitation. In addition, Ji et al. (18) found that psychological nursing intervention combined with family-like care could alleviate negative emotions and improve prognosis in patients undergoing radical mastectomy. These findings further support the need to integrate psychological support, family involvement, and individualized rehabilitation guidance into self-care management for patients with BCRL, particularly those who have undergone more extensive surgical procedures.

#### 4.2.3 Upper limb function-disability-health status

The results of this study showed that patients with BCRL generally experienced upper limb functional impairment, psychological distress, limitations in household activities, reduced mobility, and difficulties in social participation. This finding is consistent with the study by De Vrieze et al. (19). Hierarchical regression analysis further showed that upper limb function-disability-health status was significantly associated with self-care behavior. Patients with poorer upper limb function and greater daily life limitations may experience more difficulty performing self-care activities, such as limb protection, skin care, exercise, and continuous symptom management. Therefore, healthcare professionals should evaluate patients' physical, psychological, and social functioning when developing self-care support plans. Evidence-based rehabilitation guidance should be provided to relieve swelling, improve limb function, reduce psychological distress, enhance daily activity performance, and promote social participation. A comprehensive approach that combines functional rehabilitation, psychological support, and self-management education may help patients improve their self-care practices and overall health-related quality of life.

#### 4.2.4 Health beliefs

The health belief model (20) suggests that health beliefs play an important role in accepting health guidance, reducing unhealthy behaviors, and adopting health-promoting behaviors. In this study, health belief level was significantly associated with self-care behavior among patients with BCRL. This finding is consistent with previous studies in chronic disease management, including hypertension and diabetes (21, 22). Therefore, lymphedema specialists should assess patients' health beliefs during self-care management and provide individualized support. Strengthening perceived benefits, improving self-efficacy, reducing perceived barriers, and increasing cues to action may help patients translate health beliefs into sustained self-care practices.

### 4.3 Limitations

Several limitations of this study should be noted. First, this was a single-center cross-sectional study conducted in one tertiary cancer hospital, and participants were selected using convenience sampling. Therefore, the study population may not fully reflect the diversity of patients with BCRL across different regions, hospital levels, community settings, and healthcare systems. Second, although several demographic and clinical variables were collected, including education level, employment status, monthly income, and lymphedema duration, some potentially relevant confounders may not have been fully captured or adjusted for in the final regression model. For example, socioeconomic status was only indirectly reflected by variables such as education level, employment status, and monthly income, and access to lymphedema-related care, rehabilitation services, professional guidance, and patient education resources was not directly assessed. These factors may influence patients' self-care behaviors and may have introduced residual confounding. Consequently, the generalizability and interpretation of the present findings should be considered with caution. Future multicenter studies with larger and more diverse samples should include more comprehensive assessment of socioeconomic status, disease duration, healthcare access, and rehabilitation support to further validate these findings and improve external validity.

## 5 Conclusion

Self-care behavior among patients with BCRL was moderate. Age, BMI, surgical approach, upper limb function-disability-health status, and health belief level were significantly associated with self-care behavior. Because this study used a cross-sectional design, causal relationships cannot be inferred from these findings. Healthcare professionals should assess patients' upper limb function-disability-health status and health beliefs and provide individualized education, rehabilitation guidance, and self-management support. As this was a single-center exploratory study, the findings should be interpreted with caution. Future multicenter studies with larger samples and formal *a priori* sample size estimation are needed to validate these findings and provide more representative and robust evidence.

## Data Availability

The original contributions presented in the study are included in the article/supplementary material, further inquiries can be directed to the corresponding author.
